# Impact of modifiable risk factors on prediction of 10-year cardiovascular disease utilizing framingham risk score in Southwest Iran

**DOI:** 10.1186/s12872-023-03388-4

**Published:** 2023-07-18

**Authors:** Nader Saki, Hossein Babaahmadi-Rezaei, Zahra Rahimi, Maedeh Raeisizadeh, Fateme Jorfi, Faeze Seif, Bahman Cheraghian, Hossien Ghaderi-Zefrehi, Maryam Rezaei

**Affiliations:** 1 Hearing Research Center, Clinical Sciences Research Institute, Ahvaz Jundishapur University of Medical Sciences, Ahvaz, Iran; 2 Department of Clinical Biochemistry, Faculty of Medicine, Hyperlipidemia Research Center, Ahvaz Jundishapur University of Medical Sciences, Ahvaz, Iran; 3 Department of Biostatistics and Epidemiology, School of Public Health, Ahvaz Jundishapur University of Medical Sciences, Ahvaz, Iran; 4 Department of Biostatistics and Epidemiology, School of Public Health, Ahvaz Jundishapur University of Medical Sciences, Ahvaz, Iran; 5 Atherosclerosis Research Center, Ahvaz Jundishapur University of Medical Sciences, Ahvaz, Iran

**Keywords:** Cardiovascular disease, Framingham risk score, Hoveyzeh

## Abstract

**Background:**

This cohort study was conducted to examine the association between modifiable risk factors, including hypertension, smoking, physical activity, diabetes, cholesterol, and high-density lipoprotein with Framingham risk score in the prediction of 10-year-risk of cardiovascular diseases (CVD) between men and women in an Arab community of Southwest Iran, Hoveyzeh.

**Materials and methods:**

A total of 8,526 people aged 35–70 participated in this cohort study. Framingham was used to estimate the 10-year risk of CVD. Also, the linear regression models were used to assess the relationship between modifiable risk factors and the 10-year risk of CVD. Finally, the area under the receiver operating characteristic curve (AUC) was used to measure the ability of modifiable risk factors to predict the 10-year risk of CVD.

**Results:**

Our results of linear regression models showed that hypertension, smoking, PA, diabetes, cholesterol, and HDL were independently associated with the CVD risk in men and women. Also, AUC analysis showed that hypertension and diabetes have the largest AUC in men 0.841; 0.778 and in women 0.776; 0.715, respectively. However, physical activity had the highest AUC just in women 0.717.

**Conclusion:**

Hypertension and diabetes in both gender and physical activity in women are the most important determinant for the prediction of CVD risk in Hoveyzeh. Our cohort study may be useful for adopting strategies to reduce CVD progression through lifestyle changes.

## Introduction

Cardiovascular disease (CVD) is the most important cause of death in developed countries and is rising in developing countries, including Iran [1, 2]. According to the World Health Organization (WHO), annual CVD mortality will rise significantly from 17.3 million in 2008 to 22.2 million in 2030 [3]. The risk factors leading to CVD are dramatically increasing in worldwide [4]. Hypertension, smoking, diabetes, high blood lipid, unhealthy diets, physical inactivity and higher body mass index (BMI) are the most modifiable risk factors for CVD [5, 6]. Moreover, there is already considerable evidence that risk factors are inter-correlated with each other that increases CVD risk [7]. For example, the high prevalence of hypertension in older people and among individuals with diabetes is related to increased CVD mortality rates [8, 9]. Physical inactivity and smoking play a crucial role in the development of hypertension, type 2 diabetes, hyperlipidemia, and obesity that causes specific cardiovascular events [10]. Modifying one’s lifestyle is essential in improving CVD’s prognosis [11]. Accordingly, identifying associated risk factors may be beneficial for finding opportunities to prevent CVD, reducing medical costs, and saving the time needed to take preventative measures and change lifestyles [12]. Also, the high prevalence and severity of CVD require to pay more attention to old and new screening tools for better estimation. There are many techniques to predict CVD risk. The most commonly used selection technique includes the Framingham Risk Score (FRS). Several studies have been performed on the external validity of the FRS 2008 as a common and simple tool in the world. Thus, it is among the most applicable approaches to assess long-term (10-year) CVR worldwide [13, 14]. As a guideline, an FRS model can evaluate the CVD risk in North America aimed primarily the prevention. This model was achieved and presented by the Framingham prospective cohort study to evaluate the 10-year CVD risk within the 30–75years age range. By the FRS model, a multivariable function is performed. In this model, the data of HDL, smoking, and CVD risk factors are combined such as age, gender, diabetes development status, systolic blood pressure, and total cholesterol. Hence, cardiovascular risk is calculated in a specific period (e.g., 10 years) [15]. In this work, FRS is utilized as a sign of the possible advantages of prevention presented by this risk score. It identifies women and men at increased risk for future cardiovascular events. Thus, it can be effective for both the clinicians’ and patients’ decisions about preventive medical treatment and lifestyle modification as well as for patients’ education [16].

Our previous cohort study in the Arab population of Hoveyzeh showed the high prevalence of some components of CVD, including low physical activity (PA), increased blood pressure, smoking, diabetes, high cholesterol level, and low HDL level [17]. To our knowledge, previous studies in this region have not examined the association between modifiable risk factors with the 10-year high risk of CVD as a predictor in Hoveyzeh. Therefore, this research was conducted to investigate the effectiveness of modifiable risk factors in predicting the 10-year risk of CVD. Also, we identify which is the most important independent predictor of CVD risk in Hoveyzeh.

## Methods

### Study population

This population-based adult cohort study was conducted by the Prospective Epidemiological Research Studies in Iran (PERSIAN) in Hoveyzeh (Southwest Iran) from May 2016 to August 2018. The details of the Hoveyzeh cohort, a population-based cohort study, have already been published [18]. The HCS is a cohort study based on the prospective population of 10 009 adults (aged 35–70 years) recruited to evaluate NCDs in southwestern Iran. About 7772 people were eligible living in Hoveyzeh County according to the 2016 door-to-door census. Moreover, 4331 suitable people were requested to come to Susangerd bordering Hoveyzeh. Before starting the project, to increment the participation rate of the residents, several meetings were held with local trustees and authorities for familiarizing them with the benefits and steps of the study. The census was then gathered by local sanitary inspectors and Red Crescent volunteers. Hence, the needed data of all suitable individuals was recorded by trained staff. Besides, the Garmin GPSMAP 78s was used to define and record the geographical coordinates of each visited house. Trained inviters presented the invitations to the cohort site a week before the referral day. Among 12,103 eligible invited people, 8792 were enrolled in the study for step one, 982 for step two, and 235 for step three. Therefore, the study included 10 009 people with an overall response proportion of 85.16%. Of these participants, 1483 individuals were excluded due to a history of heart disease (e.g., angina, myocardial infarction, cardiovascular surgery, stroke, peripheral artery involvement, or carotid artery disease). The quality control instructions approved the protocol of the Persian cohort study. Finally, 8526 people, including 3520 men and 5006 women, were selected for analysis. The skilled interviewer completed interviewer-administrated questionnaires to assess the people’s demographic data (i.e., age, gender, education, and marital status (married or single)), cigarette smoking (non-smoker or smoker), PA (sedentary or quartile1, light or quartile2, moderate or quartile3, and vigorous or quartile4), and medical history (diseases such as diabetes, hypertension, and dyslipidemia). The presence/absence of hypertension treatment, blood pressure level, and laboratory measurements was also investigated by trained nurses. Finally, self-reported smoking, PA, and diabetes were estimated.

### Data collection

After 10–12 h of fasting, all blood samples of 25 ml (including a 7-ml clot tube and three 6-ml EDTA tubes) were drawn to measure the desired biochemical parameters. These parameters were serum fasting blood sugar (FBS), high-density lipoprotein cholesterol (HDL-C), triglyceride (TG), low-density lipoprotein cholesterol (LDL-C), and total cholesterol. All these parameters were measured using the automatic biochemistry analyzer (BT 1500) through enzymatic methods. Trained nurses performed blood pressure measurements twice (10 min apart) using a Richter hand barometer. Systolic blood pressure (SBP) ≥ 140 mm Hg and/or diastolic blood pressure (DBP) ≥ 90 mm Hg are considered hypertension. Body weight was measured using the Seca 755 Mechanical Column Scales. The measured weights were then classified as underweight < 18.5, normal 18.5-<25, overweight 25.0-<30, and obese ≥ 30 using the BMI formula (BMI (kg/m^2^) = body weight (kg) / height^2^ (m^2^)). PA was measured using self-report questionnaires. Based on the metabolic equivalent of task (MET), PA was separated into four quarters. Sedentary or Quartile 1: ≤ 33.33 METs, light or Quartile 2: 33.34–36.3 METs, moderate or Quartile 3: 36.31-39.6 METs, and vigorous or Quartile 4: ≥ 39.61 METs. The PA variable was created by summing four quartiles. Height measurements were taken barefoot using the Seca 206 Wall Mounted Height Meter.

### Framingham risk score

For the first time, the FRS was oriented by the Framingham Heart Study data to assess the 10-year risk of coronary heart disease (CHD) [5]. It was validated by multiple groups that evaluate the cerebrovascular events, peripheral artery disease, 10-year CVD risk, and heart failure as disease results for the Framingham Risk Score(2008), over CHD [6]. Though, various extensively accepted risk factors were not included in the original model for heart disease risk (1998) [5]. The present version of the FRS was developed in 2008 while the former version was presented in 2002. Age, HDL cholesterol, blood pressure, sex, LDL cholesterol, (and treatment of the patient for his/her hypertension or not), smoking, and diabetes were included in the first FRS. By modifying the improved version, dyslipidemia, hypertension treatment, age range, smoking, and total cholesterol were included [7]. The updated FRS model was calculated based on risk factors such as sex, age, total cholesterol (mmol/L or mg/dl) < 160, 160–199, 200–239, 240–279, and ≥ 280 mg/dL, HDL (mg/dl), < 40, 40–49, 50–59, and ≥ 60 mg/dL, blood pressure (mmHg) < 120, 120–129, 130–139, 140–159, and ≥ 160 mmHg, smoking (Yes/No) and the presence of diabetes (Yes/No). The CVD risk percentage is divided into three categories: low (10%), intermediate (10–20%), and high (> 20%) [15].

### Statistical methods

The IBM SPSS Statistics version 20 for Windows was used to analyze the data. Frequencies and percentages were provided for the categorical variable. Laboratory and biomedical characteristics (continuous variable) were expressed as mean ± standard deviations (SD). The characteristics of subjects were presented based on gender. In addition, the Student^’^s t-test was used to assess the independent variables. Also, Chi-squared (χ2) was used to examine associated variables based on gender. Pearson^’^s correlation coefficient was evaluated between six (continuous) variables and FRS. A univariate linear regression model was used to estimate the relationship between modifiable risk factors and 10-year CVD risk. Multiple linear regression was performed to explore factors independently associated with the 10-year CVD risk. Performing adjustments for all potential confounders revealed the association between CVD and modifiable risk factors. The potential confounding variables in this study were age and body mass index, diet, history, and antihypertensive treatment. In this respect, using linear regression models is appropriate because the effects of other variables are different at different levels of the dependent variable. Besides, the efficacy of modifiable risk factors to forecast the 10-year high risk of CVD events was evaluated using receiver operating characteristic (ROC) curve analysis. The area under the ROC curve (AUC) was statistically compared between model comparisons. An AUC of 0.7 is acceptable [19]. Finally, ROC curves were plotted and compared with Medcalc Version 2.0. Statistical significance was set at p-value < 0.05 using 2-tailed tests.

## Result

The participants of this study included 8,526 people, of which 3,520 (41.3%) were men and 5,006 (58.7%) were women, selected by the PERSIAN cohort study in Hoveyzeh [13]. The mean age of participants was 47.9 ± 9 years, with an age range of 35–70 years. The general biochemical characteristics of subjects and the data of FRS score based on gender are presented in Table 1. The frequency of PA (except Quartile 1 and Quartile 4), hypertension, diabetes, non-smoking, BMI (except normal) were higher in women than men. The men had lower serum biochemistry profiles (i.e., cholesterol, LDL, and HDL) but higher TG and FBS levels than women.

**Table 1:** Baseline characteristics of subjects based on gender

Variables	Male (n %)	Female (n%)	Total (n %)	P-Value
**Age (**± **SD)**	48.2 ± 9	47.7 ± 9	47.9 ± 9	< 0.009
**Physical activity (METs)**				
Quartile 1	1027(52.6%)	924(47.4%)	1951(22.9%)	< 0.001
Quartile 2	646(30%)	1507(70%)	2153(25.3%)	
Quartile 3	577(26.4)	1606(73.6%)	2183(25.6%)	
Quartile 4	1270(56.7%)	969(43.3%)	2239(26.3%)	
**Education**				
Illiterate	1391(26.9%)	3774(73.1%)	5165(60.6%)	< 0.001
Primary school	733(50.6%)	717(49.4%)	1450(17%)	
Secondary school	414(69%)	186(31%)	600(7%)	
High school	484(72.9%)	180(27.1%)	664(7.8%)	
University	498(77%)	149(23%)	647(7.6%)	
**High blood pressure(mmHg)**	2844(42.2%)	3897(57.8%)	6741(79.1%)	< 0.001
**Diabetes**	2812(41.1%)	4035(58.9%)	6847(80.3%)	< 0.001
**Smoking**	1368(80.3%)	336(19.7%)	1704(20%)	< 0.001
Non-smoking	2152(31.5%)	4670(68.5%)	6822(80%)	
**BMI (kg/m** ^**2**^ **)**				
Underweight	60(46.2%)	70(53.8%)	130(1.5%)	< 0.001
Normal	1000(50.9%)	966(49.1%)	1966(23.1%)	
Over weight	1508(47.1%)	1696(52.9%)	3204(37.6%)	
Obese	952(29.5%)	2274(70.5%)	3226(37.8%)	
Height (cm) (Mean ± SD)	173 ± 6.3	158.8 ± 5.6	164.7 ± 9.1	< 0.009
**Biochemical Characteristics (Mean** ± **SD)**				
TG (mg/dl)	185.8 ± 128.8	149.2 ± 92.6	164.3 ± 110.5	< 0.001
Cholesterol(mg/dl)	188.5 ± 38.7	191.7 ± 40	190.4 ± 39.5	< 0.001
HDL(mg/dl)	46 ± 10.3	53.3 ± 12.2	50.3 ± 12	< 0.001
LDL(mg/dl)	105.9 ± 31.6	108.7 ± 32.5	107.5 ± 32.2	< 0.001
FBS(mg/dl)	111.6 ± 49.1	109.3 ± 45.7	110.2 ± 47.1	< 0.027
**Framingham Risk Score category**				
< 10%	2918(82.9%)	3734(74.6%)	6652(78%)	< 0.001
10–20%	432(12.3%)	775(15.5%)	1207(14.2%)	
≥ 20%	170(4.8%)	497(9.9%)	667(7.8%)	

FBS: fasting blood sugar; HDL-C: high-density lipoprotein cholesterol; TG: triglyceride; LDL-C: low-density lipoprotein cholesterol; TC: total cholesterol; BMI: body mass index

Chi-squared (χ2) was used to examine associated variables based on gender. The Student’s t-test was used for continuous variable

The univariate linear regression model (Table 2) and associated 95%CI were considered to identify the univariate effect of the independent modifiable risk factors on the prediction of 10-year CVD risk. The results showed that hypertension, diabetes, PA, smoking, cholesterol, and HDL in men (0.088; 0.081; -0.001; 0.037; 0.005; -0.00002) and in women (0.114; 0.102; -0.007; 0.146; 0.001; -0.001) were significant univariate predictors of CVD risk, respectively.

**Table 2:** Univariate linear regression model with CVD risk determined by the FRS as dependent variable

		B	S.E	β	p-value	95%CI	
						lower	upper
Male	Constant	-0.17	0.006		0.003		
	Hypertension	0.088	0.002	0.524	< 0.001	0.069	0.077
	Diabetes	0.081	0.002	0.490	< 0.001	0.062	0.069
	Physical activity	-0.001	0.000	-0.133	< 0.001	-0.001	0.001
	Smoking	0.037	0.002	0.273	< 0.001	0.033	0.039
	Cholesterol	0.005	0.000	0.208	< 0.001	0.000	0.001
	HDL	-0.00002	0.000	0.004	< 0.001	-0.000	0.000
Female	Constant	0.062	0.010		< 0.001		
	Hypertension	0.114	0.003	0.502	< 0.001	0.085	0.093
	Diabetes	0.102	0.003	0.427	< 0.001	0.096	0.108
	Physical activity	-0.007	0.000	-0.281	< 0.001	-0.003	-0.004
	Smoking	0.146	0.005	0.385	< 0.001	0.121	0.135
	Cholesterol	0.001	0.000	0.316	< 0.001	0.100	0.110
	HDL	-0.001	0.000	-0.075	< 0.001	-0.001	0.001

Note: B: unstandardized; β: standardized; HDL: high density lipoprotein

A multiple linear regression model (Table 3) was performed to determine the significant modifiable risk factors as independent variables selected to predict CVD in 10 years (FRS) as the dependent variable. The results showed that hypertension and diabetes were associated with higher FRS (p < 0.001) in men (0.073; 0.065) and women (0.088, 0.073), respectively. In contrast, PA and HDL were inversely associated with FRS in men (-0.0004; -0.003) and women (-0.003; -0.001), respectively. Cholesterol (in men 0.003 and in women 0.001) and smoking (in men 0.036) just remained significant with FRS. Smoking in women 0.127 has a highly significant association with FRS.

**Table 3:** Multiple linear regression model with CVD risk determined by the FRS as dependent variable

		B	S.E	β	p-value	95%CI	
						lower	upper
Male	Constant	-0.010	0.006		0.003		
	Hypertension	0.073	0.002	0.438	< 0.001	0.069	0.077
	Diabetes	0.065	0.002	0.394	< 0.001	0.061	0.069
	Physical activity	-0.0004	0.000	-0.049	< 0.001	-0.001	0.000
	Smoking	0.036	0.002	0.266	< 0.001	0.033	0.039
	Cholesterol	0.003	0.000	0.197	< 0.001	0.000	0.001
	HDL	-0.003	0.000	-0.049	< 0.001	0.000	0.001
Female	Constant	0.086	0.010		< 0.001		
	Hypertension	0.088	0.002	0.387	< 0.001	0.084	0.092
	Diabetes	0.073	0.002	0.305	< 0.001	0.069	0.078
	Physical activity	-0.003	0.000	-0.120	< 0.001	-0.003	-0.002
	Smoking	0.127	0.003	0.334	< 0.001	0.120	0.133
	Cholesterol	0.001	0.000	0.267	< 0.001	0.000	0.001
	HDL	-0.001	0.001	-0.150	< 0.001	-0.002	-0.001

Note: B: unstandardized; β: standardized; HDL: high density lipoprotein

**Fig. 1:**
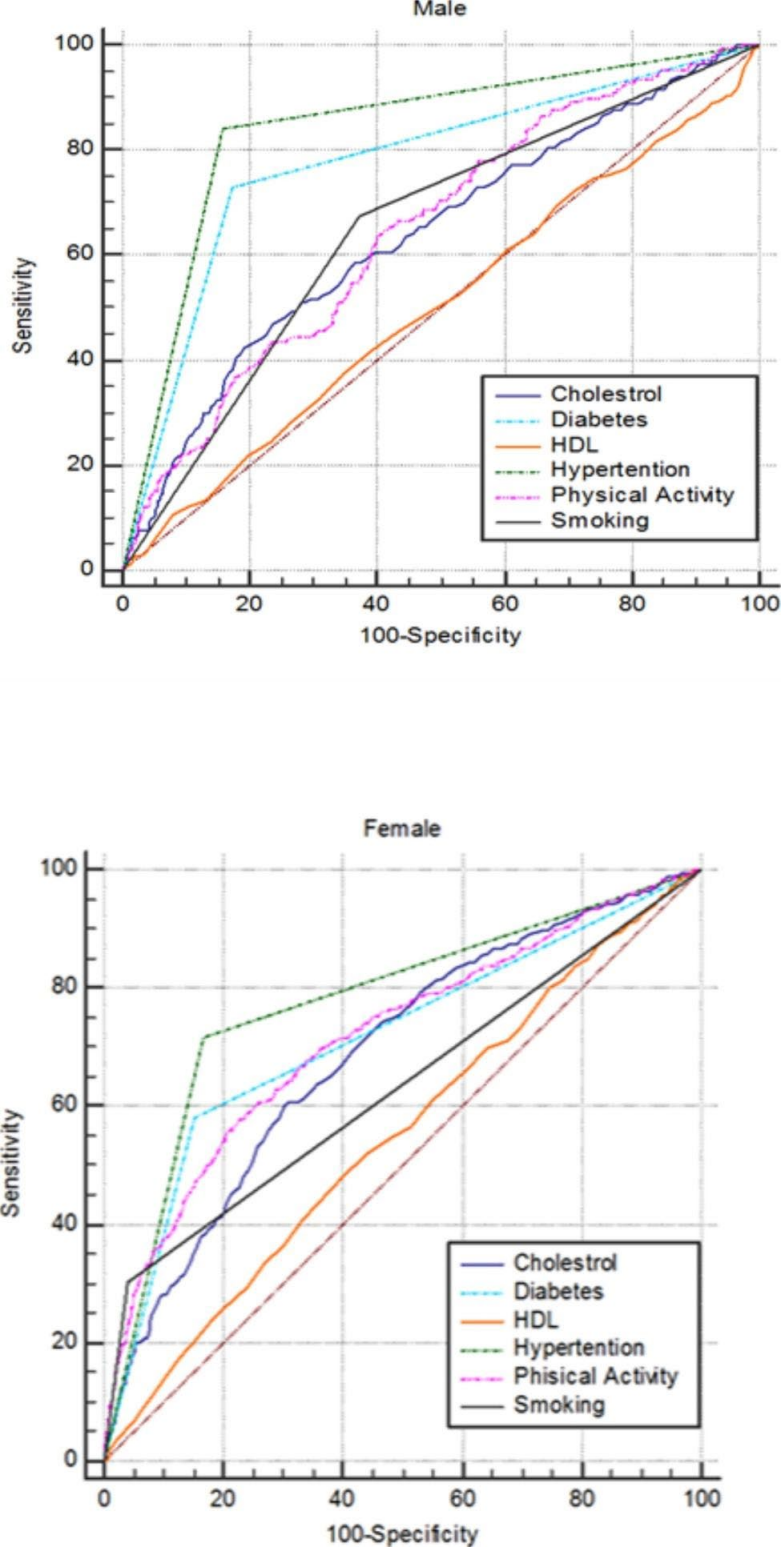
ROC curves to compare the predictive ability of modifiable risk factors for CVD risk in men and women

Figure 1 show the ROC curves for modifiable risk factors. Table 4 compares AUC and associated 95% CI of modifiable risk factors for predicting CVD. The AUC in men for hypertension, smoking, PA, diabetes, cholesterol, and HDL were (0.841; 0.651; 0.649; 0.778; 0.639; 0.502), respectively. In women, these scores were (0.776; 0.631; 0.717; 0.715; 0.688; 0.549), respectively. These data indicate that hypertension in both genders, diabetes in men, and PA in women have the largest ability to predict of CVD.

**Table 4:** Comparison of AUC and associated 95% CI of the modifiable risk factors for predicting CVD risk

Model	AUC and 95% CI	AUC and 95% CI
	Male	Female
Hypertension	0.841(0.829–0.853)	0.776(0.764–0.787)
Smoking	0.651(0.635–0.667)	0.631(0.618–0.645)
Physical ativity	0.649(0.633–0.664)	0.717(0.704 − 0.703)
Diabetes	0.778(0.174-0791)	0.715(0.702–0.728)
Cholesterol	0.639(0.623–0.655)	0.688(0.675-0.70)
HDL	0.502(0.485–0.518)	0.549(0.535–0.563)

Hypertension (SBP ≥ 140 mmHg, or DBP ≥ 90 mmHg), Smoking (yes or no), Physical activity ( min/WK), HDL and Total cholesterol (mg/dL), and Diabetes (yes or no)

## Discussion

This work aims at examining the significance of hypertension, smoking, physical activity, diabetes, cholesterol levels, and HDL levels in the prediction of the risk of developing CVD. Additionally, the study aimed to explore the association between these risk factors and the FRS. The findings indicate that hypertension, smoking, physical activity, diabetes, cholesterol levels, and HDL levels are each independently linked to the risk of developing CVD. These data demonstrate that based on regression model results, hypertension and diabetes are the main determinant of FRS. Through AUC analysis, this study also showed that hypertension and diabetes in men and women, PA only for women, have a significant ability to predict the 10-year risk of CVD from other variables. In addition, although our previous reports showed that high cholesterol levels and low HDL levels have a high prevalence of CVD, this study found that these two alone have poor predictive power for CVD risk compared with other risk factors. All of these risk factors can be modified, which means they can be changed through adopting a healthy lifestyle. This includes engaging in regular exercise, maintaining a healthy diet, refraining from smoking. Implementing these changes can effectively prevent the CVD risk [20]. The outcomes of the present work hold potential for enhancing the health and quality of life of the population in Hoveyzeh. Given the similarities in terms of ethnicity, lifestyle, race, and culture, the research findings can be generalized to a broader region encompassing southern region of Iraq and southwestern region of Iran. The results offer significant information for healthcare policymakers to devise strategies for the prevention and control of CVD. Similar our work, in Korea, a prospective cohort study was conducted to investigate the relation between CHD risk factors and estimated risk of CHD using the FRS. The study involved 15,239 men and was conducted in 2005 and 2010. The results of this study revealed significant associations between the FRS and several risk factors. These included LDL-cholesterol, hypertension, diabetes, regular exercise, and BMI. The findings indicated that these risk factors were correlated with the estimated risk of CHD as determined by the FRS [21]. In Australia, Dhaliwal and colleagues conducted a study that examined predictors of CHD deaths. The study found that smoking status, the TC to HDL-C ratio, and HDL-C were important univariate predictors of CHD deaths. This suggests that these factors play a role in influencing the risk of CHD-related mortality [22]. Rodondi et al. conducted a study for assessing the prognostic performance of the FRS, traditional risk factors, and the inclusion of additional lifestyle and laboratory variables in predicting CVD deaths. They found that systolic blood pressure, along with smoking status, TC to HDL-C ratio, HDL-C, and the FRS, were main predictors of CVD deaths. The study also investigated the utility of adding lifestyle factors such as physical activity and simple laboratory variables not included in the FRS but known to predict CHD in older adults [23]. In contrast to our study, Ruijter et al. examined the effectiveness of traditional risk factors and new biomarkers in predicting cardiovascular mortality among elderly individuals in the general population who had no previous history of CVD. The findings revealed that classic risk factors, including those incorporated in the FRS, did not accurately predict cardiovascular mortality in individuals aged 85 and above [24].

In the present study, the prevalence of hypertension is higher in women compared with men. One possible explanation for this difference between men and women is that a high incidence diabetes may increase the prevalence of hypertension in women compared with men. Some studies showed that the frequency of hypertension in women with diabetes mellitus was higher than in men [25]. In addition, women had lower education levels and less intense PA than men, which were associated with CVD risk. A cross-sectional study in Brazil showed that the prevalence of hypertension in women with low education and older age was higher than in men [26]. Moreover, in our study, the regression model results showed that hypertension increased CVD risk by 8.9% in men, which was lower than in women at 10.5%. Moreover, as measured by the ROC of hypertension, the predicting ability was high in both gender for CVD risk. In line with our findings, Vishram et al. showed that blood pressure variability forecasts CVD independent of classic cardiovascular risk factors estimated by FRS [27]. The association between CHD and its risk factors predicted by FRS in Korea was investigated by Ryoo et al. Consistent with our work, they found a considerable relation between hypertension along with other risk factors and the FRS [21]. Ramachandran et al. studied the association between the blood pressure category and incidence of CVD at baseline on follow-up for 6859 contributors in the Framingham Heart Study. Initially, the subjects were without CVD and hypertension. High-normal blood pressure is related to an increased CVD risk [28]. These results were dispelled by Framingham researchers reporting a direct association among blood pressure and cardiovascular risk irrespective of its lability [29]. Furthermore, isolated systolic hypertension was indicated as a powerful forecaster of CVD [30].

PA is an important factor in cardiovascular health [31]. In a study conducted by Arsenault et al. it was shown that women who did not do enough PA had higher CVD risk than men. This study showed that PA had a beneficial effect in lowering CVD risk [32]. Evidence in our study supports an inverse relationship between PA and CVD risk, as a one-unit increase in PA reduced CVD risk by 0.1% in men and 0.4% in women. Women with PA can have better CVD control than men. One possible explanation for this difference may be higher frequency of smoking among men than women. Gordon et al. has reported that smoking is an important factor in the ineffectiveness of exercise in decreasing heart rate response in men [33]. Similar to our results, Hamman et al. explored the association between PA and sedentary behavior as predictors of long-term CVD risk in rheumatoid arthritis (RA) patients. The finding shows that low PA and high sedentary time increase long-term CVD risk in RA [34]. Moreover, research has shown that the prevalence of CVD in women with low PA and sedentary lifestyles increased in the Saudi Arabia population [35]. However, ROC curve results show that PA alone was not effective in predicting CVD risk in men versus women. In general, attention to PA in addition to FRS may lead to improve CHD risk estimation [32]. Furthermore, a positively significant correlation was found between regular exercise and other risk factors with the FRS (by Ryoo et al.) [21]. A relationship between CHD and physical inactivity was reported in some epidemiological studies [36, 37]. The relative risk of death from CHD for sedentary individuals are high compared to active individuals [36]. Physical activity is recommended as a key element of preventative policies in adults [38]. People exercising typically encounter a lower risk of CHD [36, 39]. No information existed about physical activity at the baseline examinations for the development of this CHD risk prediction algorithm. However, among physically active people, low HDL-C levels, cigarette smoking, and diabetes are less prevalent [40–43]. Higher levels of HDL-C are normally associated with vigorous and regular exercise as a key element for decreased CHD risk [43, 44]. A study conducted in the USA investigated the relationship between physical activity and the 10-year CVD risk in individuals classified as normal weight, overweight, or obese based on the FRS. The findings indicated that engaging in any level of physical activity was related to a decreased 10-year CVD risk, regardless of weight status [45]. Additionally, a study involving Iranian women examined the impact of a 12-week exercise program on CVD risk. The results provided evidence supporting the positive impacts of exercise training in decreasing CVD risk and improving the FRS in sedentary adults [46].

Smoking, as one of the modifiable risk factors, has a crucial role in the development of CVD [47]. Studies have shown that smoking could be used to develop and improve risk prediction models for CVD occurrence. For instance, Dhaliwal et al. showed that smoking plus central obesity could be as effective as the FRS used to predict CVD and CHD [22]. The data of the regression model showed that smoking independently improved the FRS model by 3.6% in men, which was lower than in women at 12.7%. This data reveals that women are more vulnerable than men to the harmful effects of smoking. In this regard, one meta-analysis study indicated that CHD is at greater risk for female smokers than male smokers, irrespective of other risk factors [48]. Through AUC analysis, we observed that smoking had poor ability in predicting for 10-year CVD risk. According to our previous work, smoking is among the key risk CVD factors. Smoking was more common among males than females in Hoveyzeh according to our former study [17]. However, in the present work, it was revealed that female smokers have a greater risk of CVD than male smokers. The studies in Saudi are in line with our works demonstrating that the CVD risk tended to be similar in women and men. However, the individual risk factors are different for instance, HDL and diabetes mellitus levels are lower in males compared to females, smoking is more common in men, and hypertension is higher in females aged 65 years or more [35]. Dissimilar to our study, Borhanuddin et al. found that the CVD risk factors were consistently worse among men than women, particularly, based on the higher systolic blood pressure, higher prevalence of smoking status, lower HDL level, and larger diabetes comorbidity status utilizing FRS evaluation [49]. According to the Framingham Study in line with the Study of Albany Cardiovascular Health Center, smokers were at higher risk of sudden death or myocardial infarction [50].

Some studies reported that CVD risk and atherosclerosis among individuals with diabetes is 2-3-fold higher than in healthy individuals [51]. In our results, we observed that diabetes elevated CVD risk by 6.5% in men, which was lower than in women at 7.3%. The reason for this difference in the effect of diabetes on increased heart diseases in men and women may be due to the high percentage of obesity in women [52]. This study suggested that diabetes can be used as a strong predictor of another risk factor for assessing the prediction of 10-year CVD. A study in the Asia-Pacific Region reported the effect of diabetes as a major modifiable risk factor responsible for nearly half of all CVD mortality in both gender and different age groups [53]. Also, Cho et al. found that long-term diabetes is a robust predictor of CHD mortality in diabetic men [54]. In total participants, the ROC curve distribution of diabetes varied between men and women. These data suggest that diabetes has strongest ability to predict CVD risk in men of Hoveyzeh. A relationship was reported by Kannel and Macgee between impaired glucose tolerance diabetes and each of the cardiovascular sequelae, based on sex, age, and related cardiovascular risk factors in the Framingham cohort. In this work, the relative impact of diabetes was reported on peripheral vascular disease, stroke incidence, or CHD equivalent in women and men. However, the impact is greater for women for cardiac failure and cardiovascular mortality [55]. Fewer studies have been performed on the pathogenesis of CVD in diabetes, which is less affected by hypoglycemic agents [56]. Gordon et al. revealed an association between Diabetes and a lower HDL-C level with a higher CHD risk in females by study of the FRS on people aged 49-82. Furthermore, they both have strong relationships with obesity [57].

There has been considerable interest in whether various blood lipid levels alone can be used to assess 10-year CVD prediction. Our previous study showed that low level of HDL and high blood cholesterol have a high prevalence in Hoveyzeh [17]. The results of this study suggested that 10 units (mg/dL) increase in HDL level reduced CVD risk by 0.3% in men and 0.1% in women. Moreover, a 1-mmol/L increase cholesterol level elevated CVD risk by 0.3% in men, which was lower than in women at 0.1%. Based on AUC results, HDL and cholesterol alone had lowest ability to predict CVD risk in both genders. Furthermore, the evidence from our cohort study is contrary to some study about the role of HDL and cholesterol as strong predictors of CVD. Hadaegh et al. showed that total cholesterol along with other plasma lipids seemed to be an independent predictors of CVD in the Iranian population [58]. In one study, revealed that non-HDL-cholesterol was a strong predictors of CVD mortality [59]. Rodondi found that the FRS underestimates CHD risk in elder adults. Moreover, some traditional risk factors have weaker associations with CHD risk in older adults, such as total and LDL-cholesterol. Particularly, CHD events were not predicted by total cholesterol in older females [23]. The association between CHD and its risk factors predicted by the FRS was examined by Ryoo et al. in Korea. They revealed that regular exercise and HDL-cholesterol were correlated negatively with the FRS [21]. The main outcome of Borhanuddin et al. was the 10-year risk of CVD through FRS determined in terms of lipid profile associated formulae and BMI. A higher estimation of 10-year CVD risk was yielded by the BMI-based formula than the lipid profile-oriented formula in the study for both females and males [49].

More detailed and longitudinal studies on modifiable risk factors and their combination with risk scores are needed to estimate CVD risk and improve preventive management in our region. Also, long-term public health programs should be employed for changes in quality of life and reduced rates of risk factors. In addition, using the resulting data for estimating CVD risk in asymptomatic individuals is a benefit for improving our knowledge about adopting a healthy lifestyle for therapeutic changes. Public health focus on improvements in physical function, reduced rates of smoking, identifying individuals with increased blood pressure and treating them, and control of diabetes and dyslipidemia through diet regimes have positive effects on long-term Cardiovascular Health. As some risk factors were preventable and controllable, they should be considered by health policymakers to decrease the CVD prevalence in Iran. Most of these risk factors can be modified in addition to age, sex, and family history, and regular check-ups are required by people with modifiable risk factors. Practical, CVD risk can be estimated in asymptomatic people, through the resultant data to increase awareness and knowledge, as inspiration for adopting healthy lifestyles for therapeutic alterations. Further cohort and longitudinal studies are required to approve these findings among various ethnic groups. We also used a relatively better procedure for measuring biochemical factors with high-quality instrument, with lower random error and higher precision. Furthermore, our study was among Iranian, in the Gulf area, the Middle East, in which not adequate CVD papers have been published. Thus, more excellent research is required. There are also other risk factors like resting heart rate, dietary intake, novel biochemical parameters, insulin resistance, and BMI, which should be assessed in this country.

The strength of this study included a large sample size of healthy individuals. Also, we collected extensive information about anthropometric measurements, laboratory tests, and sociodemographic data from the Arab community of southwestern Iran. In addition, this article explores relationships between different cardiovascular risk factors and 10-year CVD risk in an Iranian population. This research is the first study in this population that investigated the relationship between modifiable risk factors as independent predictors of CVD. Using risk factors that have a high prevalence in the region, along with well-known measurements such as FRS, can help manage high-risk individuals by changing lifestyles. The limitations of our cohort study include selecting the age groups from ages 35 to 70 and eliminating the age ranges over 70and under 35 years. Longitudinal studies of different ethnic groups should be conducted to confirm our findings since this study was employed in an Arab community. Moreover, potentially confounding variables were possibly not adjusted for all resulting in residual confounding. Self-reported physical sitting and activity time were used in this work, which may be imprecise. Though, it was validated that the relationship with FRS scores was attenuated by the self-reported physical activity time.

The findings of the present work should be cautiously generalized to the whole of Iran as some inconsistencies can be explained by research findings in different populations by social determinants of health, lifestyles, and kind of study. Various risk estimates were considered for CVD by various models among and even within countries. Thus, an effective risk function should be nativize for each population such as geographical and ethnic characteristics, in Iran and other countries. New CVD risk markers or other strategies may be required by substantial enhancements in discrimination for risk prediction. Formerly, we revealed that smoking, hypertension, physical activity, cholesterol, diabetes, and HDL are highly prevalent. Moreover, these risk factors are related to CVD in the Hoveyzeh community. In the present work, these modifiable risk factors were used for enhanced risk prediction of CVD. Obesity and diet as well as new risk factors are other potential markers possibly enhancing CVD risk prediction in the Hoveyzeh. Future studies should be focused on the effect of novel CVD risk markers on improving risk prediction such as FRS. For current clinical use, modifiable risk factors could still predict the CVD best as the CVD risk is underestimated by FRS. Precise estimation of absolute risk is enhanced by estimated CVD utilizing these factors as independent predictors.

## Conclusion

Our findings show that smoking and physical inactivity, hypertension, diabetes, cholesterol, and HDL are all considered to be independent predictors of 10-year risk of CVD. Based on these data, hypertension and diabetes in both gender and PA in women are key determinants for preventing CVD risk. This report supports that focusing on modifiable risk factors for preventing the 10-year risk CVD may lead to a reduced cost of treatment CVD in Hoveyzeh people. Hence, our cohort study would be useful for adopting strategies to reduce CVD progression.

## Data Availability

The datasets used and/or analyzed during the current study are available from the corresponding author on reasonable request. .**Competing interests**: Not applicable.

## List of Abbreviations

CVD: Cardiovascular disease
FRS: Framingham Risk Score
SCORE: Systematic Coronary Risk Evaluation
WHO: World Health Organization
PERSIAN: Prospective Epidemiological Research Studies in Iran
FBS: Fasting blood sugar
HDL-C: High-density lipoprotein cholesterol
TG: Triglyceride
LDL-C: Low-density lipoprotein cholesterol
TC: Total cholesterol
SBP: Systolic blood pressure
DBP: Diastolic blood pressure
BMI: Body mass index
ORs: Odds ratios
